# Supplementary material for: Predicting visual function by interpreting a neuronal wiring diagram
Source: Nature. 2024 Oct 2;634(8032):113–23. doi: 10.1038/s41586-024-07953-5 (PMC11446822; doi:10.1038/s41586-024-07953-5)
Supplement: Supplementary file 6 — CRF and ERF predictions for individual TmY4 and TmY9 cells. Analogous to Supplementary Data 3, but for TmY target types. Shown are the top four monosynaptic pathways, the strongest pathway passing through each of the top ten intermediary types (ranking from Extended Data Fig. 7), and the trisynaptic pathway Tm1–TmY–Dm3–TmY (see the section entitled Prediction of spatial normalization). [file 41586_2024_7953_MOESM6_ESM.zip › DataS4/TmY9q/720575940620352135.pdf]

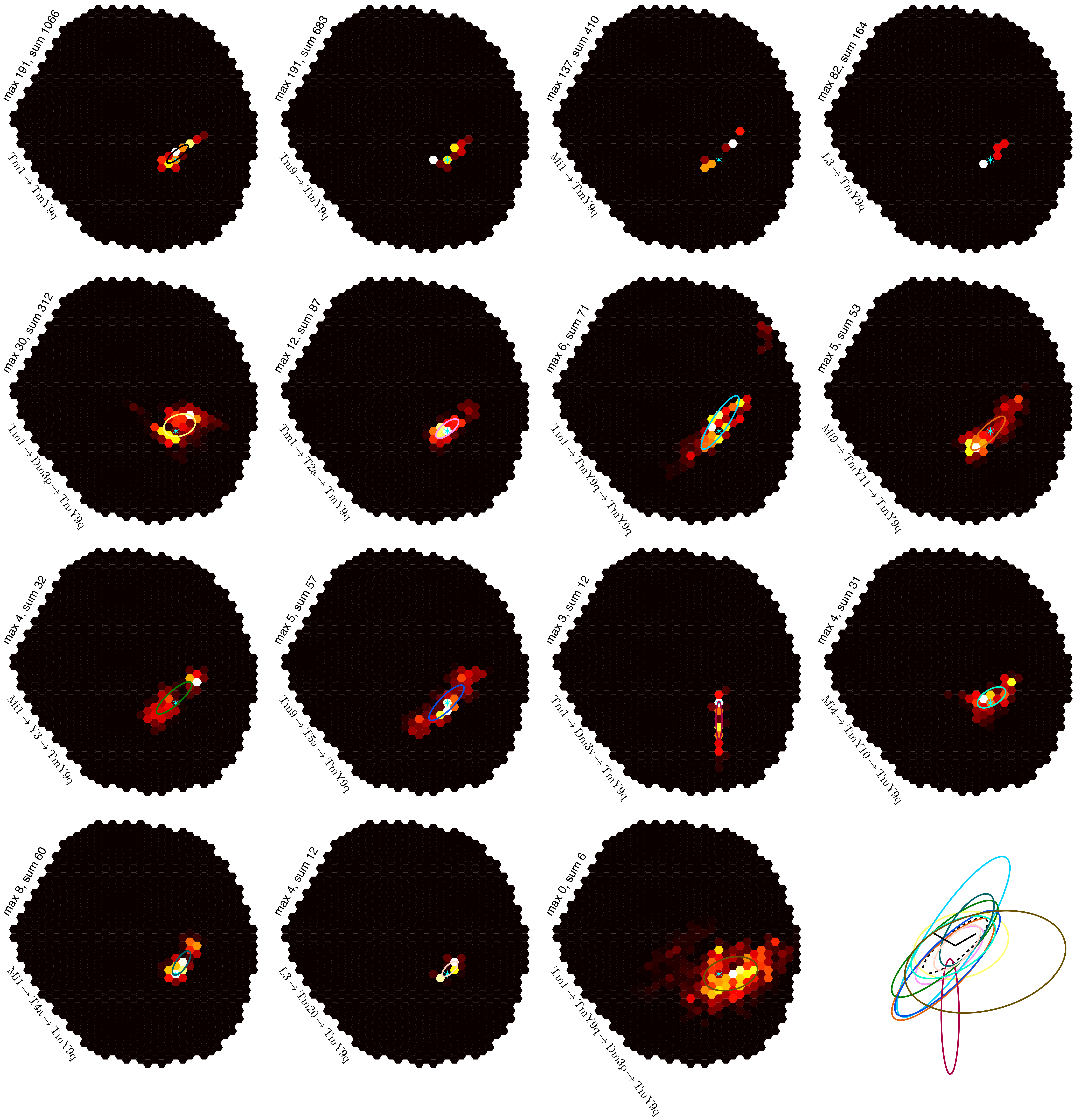
max 191, sum 1066
Tm1 → TmY9q
max 191, sum 683
Tm9 → TmY9q
max 137, sum 410
Mi1 → TmY9q
max 82, sum 164
L3 → TmY9q
max 30, sum 312
Tm1 → Dm3p → TmY9q
max 12, sum 87
Tm1 → T2a → TmY9q
max 6, sum 71
Tm1 → TmY9q → TmY9q
max 5, sum 53
Mi9 → TmY11 → TmY9q
max 4, sum 32
Mi1 → Y3 → TmY9q
max 5, sum 57
Tm9 → T5a → TmY9q
max 3, sum 12
Tm1 → Dm3v → TmY9q
max 4, sum 31
Mi4 → TmY10 → TmY9q
max 8, sum 60
Mi1 → T4a → TmY9q
max 4, sum 12
L3 → Tm20 → TmY9q
max 0, sum 6
Tm1 → TmY9q → Dm3p → TmY9q
